# Substance use patterns and negative urine opioid screen among patients on methadone treatment at a referral hospital in Nairobi, Kenya

**DOI:** 10.1371/journal.pmen.0000027

**Published:** 2024-10-14

**Authors:** Susan Wangeci Kuria, Sarah Kanana Kiburi, Jackline Ochieng, John Maina Mburu, Fredrick Owiti

**Affiliations:** 1 Mathari National Teaching and Referral Hosplital, Nairobi, Kenya; 2 Department of Psychiatry, University of Nairobi, Nairobi, Kenya; 3 Department of Medicine, Aga Khan University Hospital, Nairobi, Kenya; West Virginia University, UNITED STATES OF AMERICA

## Abstract

**Background:**

Substance use is a global health concern, with opioids contributing significantly to the disease burden. In Kenya, Medically Assisted Therapy (MAT) programs using methadone have been implemented to address opioid use disorder. Despite the effectiveness of methadone, the concurrent use of other substances remains a critical challenge. This study aimed to assess substance use patterns at enrolment and evaluate the prevalence of negative urine opioid screens among patients attending a MAT clinic at a tertiary hospital in Kenya.

**Methods:**

This retrospective cohort study analyzed data from the medical records of 713 patients enrolled in the MAT clinic between December 2014 and February 2018. Data on sociodemographic characteristics, concurrent substance use at enrolment, and urine opioid screen results at 6, 12, 18, and 24 months were collected. Multivariate analyses were performed to identify factors associated with concurrent substance use, and the likelihood of achieving negative urine opioid screens.

**Results:**

At enrolment into the MAT program, nearly all participants (712 out of 713) reported concurrent use of additional substances, with tobacco (91%) and cannabis (82.9%) being the most common. Concurrent substance use was significantly influenced by participants age, gender, education level, and route of administration of the substance. The program achieved an 81.3% retention rate at 24 months. However, the prevalence of negative urine opioid screens was lower compared to other populations, with a gradual increase from 61.3% at 6 months to 81.4% at 24 months. Notably, male patients (HR = 1.411, 95% C.I. 1.063–1.873, p = 0.01700) and those receiving higher methadone doses (HR 7.052, 95% CI 3.408–14.593, p<0.0001) were more likely to achieve negative urine opioid screens.

**Conclusions:**

This study reveals a high prevalence of concurrent substance use among patients enrolling in the MAT program in Kenya, which may affect their likelihood of achieving negative urine opioid screens. These findings underscore the need for methadone treatment programs to adopt comprehensive approaches that address all substance use disorders to improve treatment outcomes.

## Introduction

Substance use is prevalent and contributes significantly to the global burden of disease [1]. The United Nations Office on Drugs and Crime (UNODC) 2023 report showed that 296 million people had used substances globally in 2021 [1]. In Kenya, substance use is a major concern [2, 3], with a national survey revealing that over 50% of the population had used a substance at least once in their lifetime [3]. Among the various substances, opioids—defined here as both illicit opioids and prescribed medications—are associated with the highest drug related harm particularly fatal overdoses [1, 4]. This has necessitated the establishment of Medically Assisted Therapy (MAT) programs to treat Opioid Use Disorder (OUD) through opioid substitution therapy (OST) [5, 6].

In Kenya, the prevalence of opioid use is notable, being 0.1% in the general population and 4.5% among inpatients being treated for substance use disorders [7, 8]. Approved medications for OST include methadone, buprenorphine, and naltrexone [5, 6], with methadone being the most studied and widely used worldwide [5, 9, 10]. Methadone is a long-acting synthetic opioid that works by binding to the same receptors in the brain as other opioids, thereby alleviating withdrawal symptoms and reducing cravings, allowing individuals to stabilize their lives and engage in recovery [10–12]. Additionally, methadone treatment lowers the rates of infectious diseases such as Human Immunodeficiency Virus (HIV) and Hepatitis, overdose related deaths, criminal behaviours, and use of illicit drugs [10, 12, 13].

Despite the evidence that methadone is effective, challenges persist in achieving optimal treatment outcomes, particularly regarding retention rates and concurrent substance use [6, 14]. Concurrent substance use is common among patients with OUDs, with most frequent concurrent substances being alcohol, tobacco, cannabis, benzodiazepines, and amphetamines at different rates [14–17]. This multiple drug use and continued opioid use while in the MAT program negatively impact retention [6, 18, 19]. Unfortunately, most MAT programs focus on Opioids Use Disorder only, rather than a holistic approach inclusive of other substances [14, 20, 21].

Given the critical need for effective management strategies for OUD, understanding the patterns of substance use among patients in MAT is essential. Data on concurrent substance use patterns during MAT therapy in Kenya is limited. The use of methadone in the treatment of OUD in Kenya started in 2014, as part of policy to control HIV transmission associated with drug injection [22, 23]. The first MAT clinic in Kenya was established at the Mathari Teaching and Referral Hospital, and it is the largest clinic in Kenya [23]. It was sponsored by the United States President’s Emergency Plan for AIDS Relief, with implementation assistance from the University of Maryland and the United Nations Office on Drugs and Crime [22]. Currently, there are eight MAT clinics in Kenya [22–24].

This study aimed to determine the patterns of substance use at enrolment and assess the prevalence of negative urine opioid screens—defined as instances where patients test negative for illicit opioids, indicating adherence to treatment—among patients attending a MAT clinic at a national referral hospital in Nairobi, Kenya. Specifically, we hypothesized that patients enrolled at the MAT clinic in Kenya exhibit high rates of concurrent substance use, influenced by a variety of sociodemographic and clinical factors. Additionally, we posited that concurrent use of substances alongside opioids at enrolment would adversely affect the likelihood of achieving negative urine opioid screens during treatment, thereby hindering overall treatment success.

## Methodology

### Study design

This was a retrospective cohort study that involved abstraction of data for patients enrolled in methadone treatment from December 2014 to February 2020. Each participant’s data was gathered for a maximum of 24 months after the patient enrolled in the program.

### Study site

The study was conducted at the MAT clinic at Mathari National Teaching and Referral Hospital in Nairobi (MNTRH), Kenya. The catchment area of the clinic includes most parts of Nairobi City—a cosmopolitan city, as the facility is located approximately 5 kilometres from Nairobi City Centre. Patients are referred to the facility from drop-in centres after receiving psychosocial education, support, and education on other harm reduction practices like not sharing needles or syringes, no flushing, and safe sex, among others. This is in line with the national guidelines on MAT treatment [25]. All patients receive methadone oral syrup formulation, which is dispensed daily as direct observation therapy to deter diversion.

### Study population and sample size

We conducted a census of the 727 patients enrolled from December 2014 to February 2018. All the participants were adults aged 18 years and older. The last period of February 2018 was informed by the COVID-19 pandemic getting to Kenya in early March 2020. According to the Global State of Harm Reduction report, the pandemic significantly affected the operations of MAT clinics worldwide [26]. From March 2020 to the time of data collection (July 31st, 2021), the clinic did not enrol any new patients or conduct random urine drug screening tests. As a result, for the patients who were enrolled in February 2018, we were able to get their 24-month data until February 2020.

### Recruitment and data collection procedures

The medical records of the patients enrolled from December 2014 to February 2018 were retrieved based on the clinic’s register. The MAT clinic at MNTRH uses enrolment forms adopted from the Substance Abuse and Mental Health Services Administration (SAMHSA). Researcher-designed sociodemographic and data collection forms were used to collect data. The socio-demographic data collected from the medical records included: age, gender, education level, marital status, occupation, housing status, and legal status.

Other data collected included the age at which the patient first used opioids; duration of use; severity of the Opioid Use Disorder; other substances at enrolment; and urine drug screen results at 6 months, 12 months, 18 months, and 24 months. Patients self-reported their substance use during enrolment in the MAT clinic. All the patients underwent a confirmatory urine drug screen at enrolment. However, not all self-reported substances could be confirmed with a urine drug screen (e.g., alcohol, glue, benzhexol, etc.). To prevent adulteration or cheating, patients were partially supervised while collecting urine samples in sterile urine bottles, according to the standard operating procedures laid out in the clinic. Thereafter, a qualified laboratory technician conducts a urine drug screen using a dipstick technique, with the results being interpreted after five minutes. During the study period, the available drug screen kit tested for ten substances: amphetamines, barbiturates, benzodiazepines, cocaine, tetrahydrocannabinol, methamphetamine, methadone, morphine, opiate, and tricyclic antidepressants. According to SAMHSA’s recommendations, patients in the program had to undergo routine monthly drug tests [27]. However, there were no clear criteria for scheduling follow-up drug screens, and urine drug screen kits frequently ran out of stock as the number of patients increased, leading to periods when testing could not be conducted.

### Pre-testing of the study instruments

We conducted a pilot study at the MNTRH MAT clinic prior to the main study to evaluate the validity and reliability of the study tool. A sample size of 20 files was used. The pre-test assisted with assessing the accuracy, clarity, and feasibility of the main study in terms of costs and other logistics.

### Variables

The independent variable was concurrent use of substances at enrolment, referring to whether the patient used any other substances other than opioids at entry into the MAT program. The dependent variable was the prevalence of negative urine opioid screens while in the MAT program. The mediating variables included socio-demographic factors (age, sex, education level, employment status, and marital status), and clinical variables (including comorbid medical illness, age at first use of opioids, duration of opioid use prior to enrolment to MAT program, and maximum dose of methadone administered).

### Ethical considerations/ ethics statement

Approval to conduct the study was granted by the Ethics and Research Committee of Kenyatta National Hospital-University of Nairobi (KNH-ERC/A/125), and the Mathari National Teaching and Referral Hospital Research Committee.

### Data analysis

The data were entered into Microsoft Excel, cleaned, and exported to the Statistical Analysis for Data Science (STATA) version 18.3.10 software for analysis. We used descriptive statistics to describe the participant’s socio-demographic characteristics, patterns of substance use, and urine opioid drug screen at months 6, 12, 18, and 24. Bivariate analysis was performed for each concurrent substance used at enrolment, to identify the relationships between concurrent substance use, and socio-demographic and clinical variables (S1 Table). Variables that displayed an association with outcome at bivariate level, with a significance level of p<0.1, were included in a multivariate logistic regression model to identify independent predictors of use of each substance. In this model, we accounted for potential confounders by including all relevant sociodemographic and clinical factors that exhibited significant associations in the bivariate analysis. Adjusted odds ratios with 95% confidence intervals were calculated to quantify the strength and significance of these associations, with a p-value of less than 0.05 considered statistically significant.

For the analysis of urine opioid screen tests over the four-time points, cox regression modelling was used to identify socio-demographic and clinical predictors of time to a negative screen opioid test. In this study, the event of interest (the negative opioid screen) can occur multiple times and is described as a recurrent event. To manage the phenomenon of initial negative tests followed by positive tests, we implemented a robust variance estimator to account for the correlation between repeated measures on the same individual, ensuring that our model accurately reflected the independence of each event. Additionally, we assessed the proportional hazards assumption for the Cox model to ensure that the relationships between the predictors and the time to the event were consistent over time.

## Results

**Fig 1:**
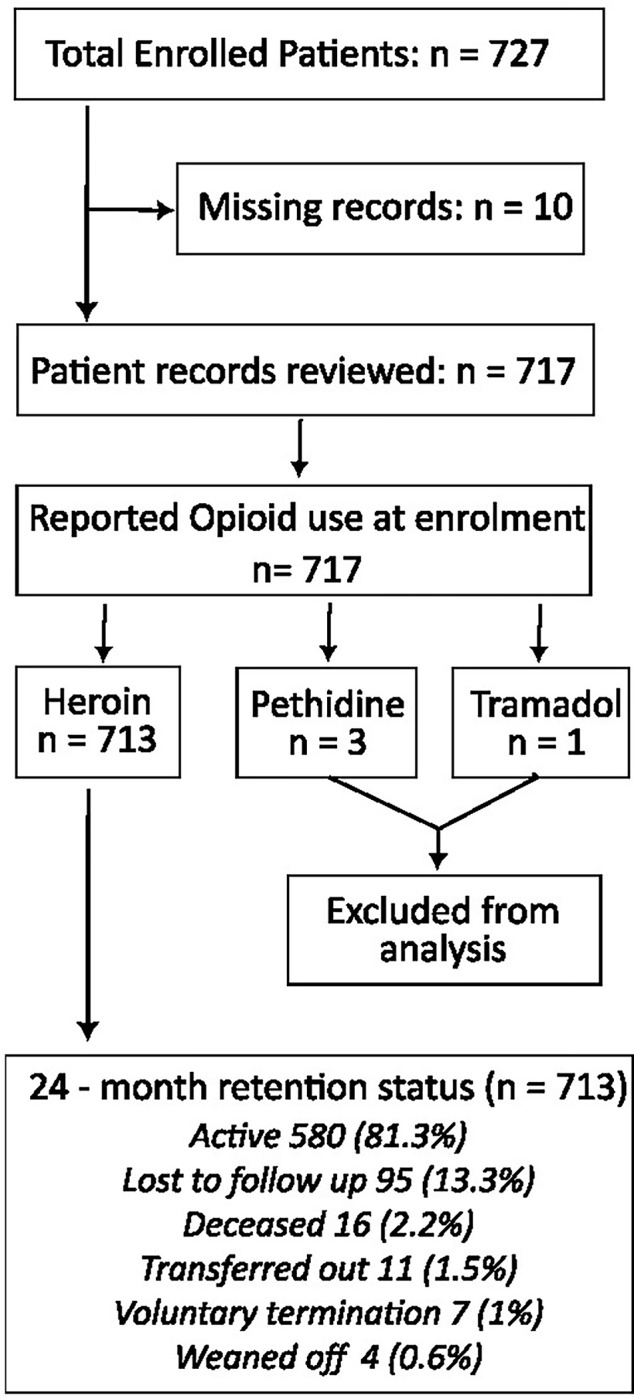
Study flow chart. From December 2014 to February 2018, 727 patients enrolled in the MAT clinic. Ten files were missing, leaving 717 files for review. Among these, the opioids reported at enrolment were heroin in 713 cases, pethidine in 3 cases, and tramadol in 1 case. The files of patients using pethidine and tramadol were then excluded as the number was too low to get power for analysis. The ultimate analysis was conducted on 713 patients’ files.

From December 2014 to February 2018, 727 patients enrolled in the MAT clinic at Mathari National Teaching and Referral Hospital (MNTRH). Ten files were missing, leaving 717 files for review. Among these, the opioids reported at enrolment were heroin in 713 cases (99.4%), pethidine in 3 cases (0.42%), and tramadol in 1 case (0.14%) as shown in Fig 1. The files of patients using pethidine and tramadol were then excluded as the number was too low to get power for analysis. The ultimate analysis was conducted on 713 patients’ files.

### Socio-demographic characteristics of study participants

Table 1 provides sociodemographic characteristics, clinical profiles of the participants at enrolment including the maximum methadone dose administered while in the program, and their retention status at 24 months. Majority of the participants were aged 26–35 years, with a mean of 34.3 ± 8.6 years (range:18–78 years). Most participants were males (85.7%), had education level of primary school and below (48.8%), were single, separated, or divorced (78.4%), and had ever been arrested (64.2%). Additionally, most participants began using opioids between the ages of 18–25 years (55.5%), had used opioids for less than 8 years, required methadone dose of 101-150mg (43.2%), and were active in the MAT program for at least 24 months (81.3%). Two hundred and sixty-one patients (36.6%) had medical comorbidities, with the most common being HIV and Hepatitis C.

**Table 1:** Sociodemographic characteristics, clinical profile, maximum methadone dose, and retention status at 24 months.

Variable	Frequency (N = 713)	Percentage (%)
Age in years		
18–25	118	16.5
26–35	312	43.8
36–50	253	35.5
51 and above	30	4.2
Gender		
Female	102	14.3
Male	611	85.7
Education Level		
Primary and Below	348	48.8
Secondary	290	40.7
Tertiary	75	10.5
Marital status		
Married	154	21.6
Single/Separated/ Widowed	559	78.4
Occupation		
Employed	424	59.5
Self-employed	94	13.2
Un-employed	195	27.3
Housing		
Homeless	120	16.8
Lived with Friends & Relatives	159	22.3
Lived in Own or Rental House	238	33.4
Unstable	196	27.5
Legal History		
No	255	35.8
Yes	458	64.2
Age at first use in years		
Below 18	148	20.8
18–25	396	55.5
Above 25	169	23.7
Duration of use in years		
Below 8	258	36.2
8–15	247	34.6
Above 15	208	29.2
Route of administration		
Intravenous	398	55.8
Smoking	158	22.2
Both	157	22.0
Medical comorbidity		
HIV	107	15.0
Hepatitis C	81	11.4
Syphilis	44	6.2
Hypertension	5	0.7
Hepatitis B	13	1.8
Asthmatic	9	1.3
Tuberculosis	2	0.3
Maximum methadone dose (mg)		
<50	49	6.9
50–100	217	30.4
101–150	308	43.2
151–200	116	16.3
>200	23	3.2
Retention status at 24 months		
Active	580	81.3
Lost to follow up	95	13.3
Deceased	16	2.2
Transferred out	11	1.5
Voluntary termination	7	1.0
Weaned off	4	0.6

### Concurrent substance use at enrolment into MAT program

Most participants (712 out of 713) concurrently used other substances besides opioids at enrolment in the MAT program. The most commonly used additional substances were tobacco (91%) and cannabis (82.9%). Other substances used were benzodiazepines (51.5%), alcohol (20.6%), khat (10.7%), benzhexol (4.9%), barbiturates (0.6%), cocaine (2.7%), glue (0.3%), and chlorpromazine (0.1%). Most participants used two (34.4%) or three (34.8%) other substances (Table 2).

**Table 2:** Number of concurrent substances used.

Number of Substances	N	%	95% C.I	
None	1	0.1	0.0	0.4
One	87	12.2	10.0	14.9
Two	245	34.4	30.9	37.7
Three	248	34.8	31.1	38.1
Four	97	13.6	11.1	16.4
Five	29	4.1	2.7	5.6
Six	5	0.7	0.1	1.3
Seven	1	0.1	0.0	0.4

### Factors associated with concurrent substance use at enrolment

Multivariate analysis revealed that concurrent use of other substances at enrolment into the MAT program was associated with several factors including age, gender, level of education, occupation, legal status, age at first use of opioids, and route of administration (Table 3). Marital status, housing, and duration of opioid use were not statistically significant. Regarding age, for each additional year, the likelihood of using cannabis decreased by 67.9% (adjusted odds ratio (aOR) 0.32, 95% CI 0.110–0.932). Conversely, every one-year increase in age was associated with two-fold increase in the odds of alcohol consumption (aOR 2.40, 95% CI 1.298–4.423). In terms of gender: males were 45.6% less likely to use khat compared to females (a.O.R. 0.54, 95% CI 0.301–0.984). The level of education also played a significant role. Participants with a secondary level of education had a 49.1% reduced likelihood of using tobacco (aOR 0.49, 95% CI 0.269–0.899), while those with a tertiary level of education had a higher likelihood of using cocaine (aOR 4.20, 95% CI 1.070–16.473).

**Table 3:** Factors associated with substance use in multivariate analysis.

Variable	Category	Cannabis	Tobacco	Alcohol	Cocaine	Benzodiazepines	Khat	Benzhexol
		a.O.R (95% C.I)	a.O.R (95% C.I)	a.O.R (95% C.I)	a.O.R (95% C.I)	a.O.R (95% C.I)	a.O.R (95% C.I)	a.O.R(95% C.I)
Age	18–25	Ref.	--	Ref.	Ref.	Ref.	-	-
	26–35	0.85(0.43; 1.68)	--	1.63(0.90; 2.96)	4.31(0.42; 43.89)	0.93(0.60; 1.46)	-	-
	36–50	0.76(0.35; 1.64)	-	2.40(1.30; 4.42)**	10.65(0.69; 164.86)	1.02(0.63; 1.65)	-	-
	51 and above	0.32(0.11; 0.93)*	-	1.53(0.50; 4.68)	10.65(0.69; 164.86)	0.33(0.13; 0.88)*	-	-
Gender	Female	-	Ref.	Ref.	-	-	Ref.	-
	Male	-	1.95(0.97; 3.92)	0.63(0.37; 1.06)	-	-	0.54(0.30; 0.98)*	-
Education Level	Primary and Below	-	Ref.	-	Ref.	-	-	Ref.
	Secondary	-	0.49(0.27; 0.90)*	-	1.51(0.51; 4.42)	-	-	0.99(0.49; 2.02)
	Tertiary	-	0.86(0.33; 2.29)	-	4.20(1.07; 16.47)*	-	-	0.23(0.03; 1.76)
Occupation	Employed	-	Ref.	-	-	-	-	Ref.
	Self-employed	-	2.73(0.87; 8.56)	-	-	-	-	0.57(0.13; 2.53)
	Un-employed	-	0.92(0.49; 1.74)	-	-	-	-	2.48(1.21; 5.09)*
Legal History	No	Ref.	Ref.	-	-	-	Ref.	-
	Yes	0.81(0.52; 1.24)	3.39(1.89; 6.07)***	-	-	-	0.53(0.33; 0.86)**	-
Age at first use	<18	-	-	-	Ref.	Ref.	-	-
	18–25	-	-	-	0.25(0.08; 0.80)*	0.68(0.46; 1.02)	-	-
	25+	-	-	-	0.10(0.01; 0.81)*	0.61(0.37; 0.99)*	-	-
Route of opioid administration	Intravenous	Ref.	Ref.	Ref.	Ref.	-	Ref.	Ref.
	Smoking	2.06(1.18; 3.61)**	0.466(0.231; 0.939)*	1.831(1.162; 2.885)**	0.600(0.161; 2.244)	-	1.538(0.857; 2.761)	0.334(0.114; 0.983)*
	Both	2.06(1.19; 3.58)**	0.38(0.19; 0.73)**	1.16(0.72; 1.86)	0.29(0.06; 1.39)	-	1.33(0.73; 2.44)	0.37(0.13; 1.07)
Maximum Dose of methadone	< = 50	-	Ref.	Ref.	Ref.	Ref.	-	-
	50–100	-	1.92(0.76; 4.85)	3.32(1.24; 8.89)*	0.38(0.09; 1.64)	0.75(0.39; 1.44)	-	-
	100–150	-	2.68(1.08; 6.68)*	2.49(0.94; 6.59)	0.17(0.04; 0.80)*	0.67(0.36; 1.27)	-	-
	151–200	-	2.23(0.78; 6.38)	1.38(0.47; 4.05)	0.23(0.04; 1.30)	0.52(0.26; 1.06)	-	-
	>200	-	5.10(0.50; 52.49)	0.84(0.15; 4.75)	0.42(0.03; 5.34)	1.18(0.41; 3.38)	-	-

Note

*P<0.05

**P<0.01

***P<0.001;—Not statistically significant in bivariate analysis; a.O.R-Adjusted Odds Ratio

### Prevalence of negative urine opioid screens while in the MAT program

**Fig 2:**
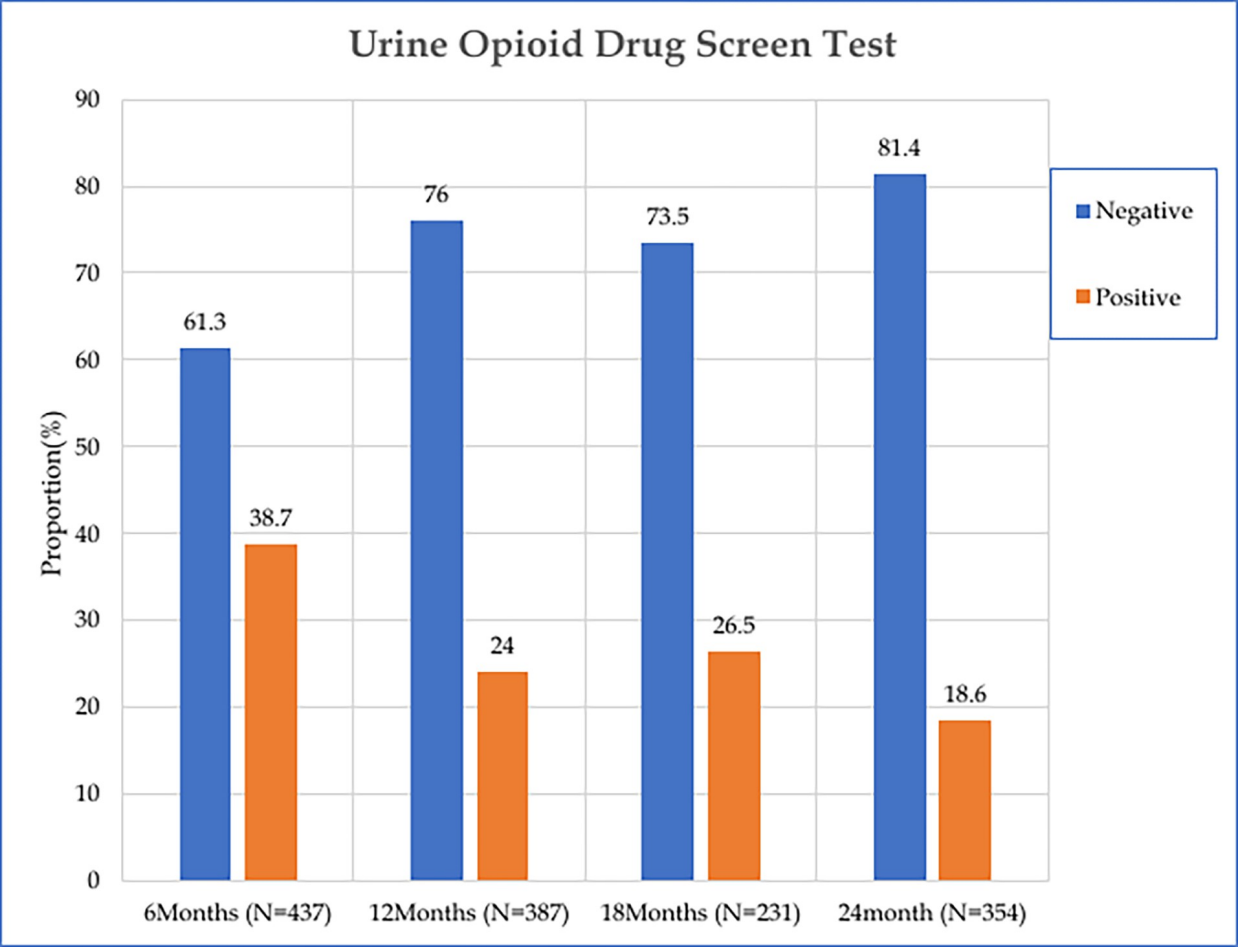
Urine opioid screens. Over the 24-month period, the prevalence of negative urine opioid screens progressively increased from 61.3% at 6 months to 81.4% at 24 months.

Over the 24-month period, the prevalence of negative urine opioid screens progressively increased from 61.3% at 6 months to 81.4% at 24 months (Fig 2). Significant factors associated with achieving a negative urine opioid screen following the initiation of methadone treatment included the patient’s gender and the maximum dose of methadone administered (Table 4). Males were 1.4 times more likely than females to have a negative urine opioid screen after initiating methadone treatment (HR = 1.411, 95% C.I. 1.063–1.873, p = 0.01700). Methadone doses exceeding 50 mg were significantly associated with an increased likelihood of a negative urine opioid screen, with the highest hazard ratios observed for doses 100–150 mg (HR 7.052, 95% C.I. 3.408–14.593). Other variables did not show statistically significant differences in achieving a negative urine opioid screen (Table 4).

**Table 4:** Factors associated with time to cease using opioids (negative urine opioid screen) after initiation.

Variable	Hazard ratio (95% C.I)	Sig.
Age in years		
18–25	Ref.	0.4730
26–35	1.125(0.816; 1.551)	0.4570
36–50	1.177(0.767; 1.805)	0.7810
51 and above	0.908(0.461; 1.790)	
Gender		
Female	Ref.	**0.01700**
Male	1.411(1.063; 1.873)	
Education Level		
Primary and Below	Ref.	0.28600
Secondary	0.902(0.745; 1.091)	0.48400
Tertiary	0.896(0.658; 1.219)	
Marital Status		
Married	Ref.	
Single/Separated/Widowed	1.114(0.885; 1.404)	0.35800
Occupation		
Employed	Ref.	
Self-employed	1.217(0.930; 1.592)	0.15300
Un-employed	1.069(0.865; 1.321)	0.53500
Housing		
Homeless	Ref.	
Friends & Relatives	1.081(0.812; 1.440)	0.59300
Own House/ Rents	1.217(0.917; 1.615)	0.17500
Unstable	1.075(0.810; 1.428)	0.61600
Legal History		
No	Ref.	
Yes	0.933(0.773; 1.125)	0.46700
Age at first use		
<18	Ref.	
18–25	1.125(0.870; 1.454)	0.37100
25+	1.237(0.859; 1.781)	0.25200
Duration of use		
<8 Years	Ref.	
8–15 yrs	1.069(0.832; 1.374)	0.60300
>15 yrs	1.228(0.864; 1.746)	0.25300
Route of Administration		
Intravenous	Ref.	
Smoking	1.037(0.826; 1.303)	0.75200
Both	1.052(0.841; 1.316)	0.65600
Maximum Dose		
< = 50	Ref.	
50–100	5.032(2.454; 10.314)	**<0.0001**
100–150	8.047(3.964; 16.336)	**<0.0001**
151–200	7.052(3.408; 14.593)	**<0.0001**
>200	7.114(3.062; 16.528)	**<0.0001**

## Discussion

This study aimed at examining the substance use patterns at enrolment into MAT program and factors associated with treatment outcomes, as evidenced by a negative urine opioid screen, among patients enrolled in the methadone treatment program at a tertiary hospital in Kenya. The findings highlight key insights into the sociodemographic characteristics, concurrent substance use, and factors influencing treatment success in this cohort.

### Significant socio-demographic characteristics of study participants

Most participants in this study were young, with a mean age of 34.3 years. This is comparable to other studies that have reported a predominance of young to middle aged individuals usually in the age group 30–40 years [13, 21, 28–31]. This is a worrying trend, as this age group is expected to be the most productive in society. Additionally, most participants in this study (55.5%) began using opioids between the ages of 18–25 years. This suggests that early adulthood is a key period for initiation of opioid use, therefore the need for preventive measures targeting this age group [31]. The relatively shorter duration of opioid use (less than 8 years) before enrolling into the MAT program indicates increased willingness to seek help among the younger individuals.

Most participants in this study were males (85.7%). This gender disparity is consistent with most findings from other studies [13, 21, 28, 30, 32]. Generally, men are more likely than women to engage in risky behaviours including use of opioids. Although there were fewer females in the current study, recent studies have reported an increasing number of women with OUDs due to risk factors such as trauma, domestic violence, and depression [15, 33]. This calls for gender specific targeted therapies to address this concern as the predisposing factors to OUDs may be gender specific.

Our findings indicate that family-social factors could be key drivers of OUDs. Most participants (78.4%) were separated, single, or divorced. Previous studies have reported mixed results [13, 21, 28, 32]. The results in this study could be due to the negative impacts of substance use disorders, which can lead to disintegration of the family unit [34, 35]. This underscores the need for integrating comprehensive treatment strategies which may involve family members of individuals with substance use, such as behavioural couple therapy [35, 36].

Educational attainment was generally low among the participants in this study, with nearly half (48.8%) having primary school or below level of education. Low education levels are associated with fewer employment opportunities, contributing to economic instability and continued substance use [37, 38]. These economic challenges may predispose to criminal behaviours. As was observed in this study, majority of participants had legal histories, with 64.2% having been arrested at some point. This highlights a cohort with significant social and legal challenges. There is need for integrated treatment approaches that address both substance use and its legal ramifications. Lack of educational attainment can limit access to information on the risks associated with opioid use leading to a vicious cycle of continued drug use and engaging in criminal activities [38, 39]. This demonstrates the need for incorporating educational and vocational training into treatment programs for economic empowerment of the participants [40].

### Concurrent substance use

Nearly all the participants (99.9%) in this study reported using additional substances along with opioids at enrolment in the MAT program. Notably, a significant portion of participants used two (34.4%) or three (34.8%) other substances. Concurrent substance use has been reported in previous studies [13, 16, 28, 31, 41, 42], and highlights the need for integrated treatment programs that can effectively address multiple substance dependencies simultaneously [30].

In the current study, tobacco (91%) and cannabis (82.9%) were the most used substances. The specific additional substance(s) used may vary depending on geographic location. Studies done in Vietnam and Australia showed the most common substance was tobacco [21, 43]. In contrast, studies done in Finland and the USA showed methamphetamine and benzodiazepine as the most common substances [15, 19]. Other studies have reported findings that are different from our study [13, 21, 28, 31, 41, 42]. The variations in patterns of substance use in these studies could be attributed to various factors, such as study design, substance availability, national legislations, and socioeconomic status.

According to the gateway theory, consuming a less potent drug with a lower risk of addiction increases the likelihood of using more powerful and potentially dangerous substances [44]. Cannabis and tobacco, which were the most commonly co-used substances in this study, are regarded as gateway drugs into opioid use [20, 44, 45]. The nicotine in tobacco binds nicotinic acetylcholine receptors in the central nervous system and stimulates the release of dopamine in the nucleus accumbens, which enhances the euphoric effects produced by other drugs [46, 47].

Several factors were identified as being associated with concurrent substance use using multivariate analysis. Age, gender, and educational level were all significant predictors of substance use. For example, younger people were more likely to use cannabis, whereas older people were more likely to consume alcohol. This implies that substance use habits may change over time, necessitating age-appropriate interventions. Furthermore, in the current study, males were less likely to use khat than females and higher education levels were associated with decreased tobacco usage but increased risk of cocaine use. These findings suggest that demographic factors influence concurrent substance use patterns, and tailored interventions based on these features may be more effective.

### Urine opioid drug screen among opioid users attending MAT

Urine opioid drug screens are critical for monitoring patients in methadone maintenance treatment (MAT) programs. This study found a significant increase in the proportion of patients who had a negative urine opioid screen, from 61.3% at six months, to 76% at 12 months, and 81.4% at 24 months. This upward trend underscores the effectiveness of methadone maintenance therapy in reducing opioid use over time. Previous studies have also reported increasing rates of negative urine screens among patients in MAT [15, 32, 48]. However, when compared to other studies, our findings reveal lower rates of negative urine opioid screens. Studies done in the US, Malaysia, and China reported higher rates of negative urine opioid screens, ranging from 89.6% to 97.6% at 12 months [15, 32, 49] and up to 98.6% at 24 months [32].

The reasons for lower negative urine opioid screens reported in the current study could be multifaceted. Most of the participants in the study had lower educational attainment and unstable family relations, which are indicative of broader socio-economic challenges. These socio-economic challenges can lead to stress and anxiety, potentially increasing the likelihood of relapse [50]. Second, the extremely high prevalence of concurrent substance use (99.9%) among our study participants presents a significant barrier to achieving negative urine opioid screens. The most used substances were tobacco and cannabis, which are known to be challenging to quit and may interfere with the treatment of opioid dependence [46]. Additionally, a significant proportion of the participants (36.6) had significant chronic medical comorbidities. The additional burden of managing these conditions in addition to OUD can be complex [51], potentially affecting adherence to the MAT program.

Frequent drug testing plays a key role in achieving opioid abstinence in patients undergoing MAT therapy as it helps identify ongoing substance use early, allowing for timely intervention and treatment adjustments [52]. It also serves as a motivational tool, encouraging patients to remain compliant with their treatment plans. The Substance Abuse and Mental Health Services Administration (SAMHSA) recommends conducting random drug screens at least once per month for patients attending MAT clinics [27]. In the current study, there were no clear criteria for scheduling the follow-up drug screens and the urine drug screen tests frequently ran out of stock. These factors could have contributed to the lower rates of negative urine opioid screens compared to other studies, and call for the establishment of clear protocols for drug screening and ensuring supplies for the screening tests are available in the MAT programs.

Our study identified patients’ gender and the maximum dose of methadone administered as significant factors associated with achieving negative urine opioid screens. Our findings indicate that males are 1.4 times more likely to have a negative urine opioid drug screen than women after initiating methadone treatment. Previous studies have reported gender differences on treatment outcomes of patients on methadone maintenance treatment for OUDs [9, 53, 54]. Women face unique challenges in accessing and remaining engaged in the treatment of OUDs such as stigma, childcare responsibilities and social support deficits [53]. Biological factors also play a role in the gender differences observed in treatment outcomes. Women may metabolize drugs differently than men, leading to variations in how they respond to medications used in MAT [9, 53, 54]. This underscores the need to consider gender-specific factors when designing and implementing treatment programs for OUD.

In the current study, patients receiving methadone doses exceeding 50 mg demonstrated a greater probability of testing negative for opioids, with the highest hazard ratios observed for those on doses between 100–150 mg. The findings from this study align with existing literature that supports the notion that appropriate methadone dosing is crucial for successful treatment outcomes [55, 56]. Optimal methadone dosing is essential for successful treatment outcomes. Methadone is a long-acting synthetic opioid that works by binding to the same receptors in the brain as other opioids, thereby alleviating withdrawal symptoms and reducing cravings [11, 12]. Patients on optimal doses are often better equipped to manage their cravings and withdrawal symptoms, which can reduce the likelihood of relapse and illicit opioid use, and allows them to function without the euphoric effects associated with other opioids [11, 12, 55]. This is particularly important in a population where concurrent substance use is prevalent, as seen in this study. This calls for personalised strategies, including ongoing monitoring, and methadone dose adjustment to optimize treatment outcomes [18].

### Retention rates in the MAT program

Retention rates in MAT programs are key indicators of treatment success as they reflect patient’s ability to remain engaged in their recovery process. The retention rate observed in our study was relatively high, with 81.3% of participants remaining active in the MAT program at 24 months. Retention rates reported in previous studies vary, ranging from 37% to 70% at 12 months [5, 6], and falling to 35% at 2 years [57]. However, while the retention rates were encouraging, they should be interpreted in the context of observed negative urine opioid screens. Although the proportion of participants achieving negative screens increased over time, these rates were lower than those reported in other populations. This discrepancy suggests that while patients may remain in treatment, there may be challenges related to achieving complete abstinence from opioids. Further studies are required to explore these challenges with the ultimate goal of improving outcomes.

## Strengths and limitations

The strength of this study includes the comprehensive and longitudinal analysis of a large cohort of 717 patients enrolled in the MAT program. This study provides robust data on the sociodemographic characteristics, substance use patterns, and treatment outcomes, offering valuable insights into the effectiveness of methadone maintenance therapy. Additionally, the identification of significant factors associated with treatment outcomes contributes to a deeper understanding of the complexities of opioid use disorder. This study highlights the need for tailored interventions and integrated treatment approaches, thereby informing future practices and policies in substance use treatment within similar contexts.

This study had some limitations that should be considered when interpreting the results. Its retrospective design, which may introduce biases related to incomplete or inaccurate medical records. The exclusion of patients using pethidine and tramadol due to small numbers may limit the generalizability of our findings to all opioid users. Additionally, this study was conducted in a single facility, and this may limit the generalizability of the results to other populations or healthcare contexts. Furthermore, the reliance on self-reported data for certain variables may introduce bias or inaccuracies.

## Conclusion

This study highlights the high prevalence of concurrent substance use among patients enrolling in methadone maintenance therapy at a tertiary hospital in Kenya, with nearly all the participants reporting the use of additional substances. Various sociodemographic and clinical variables influenced the use of these substances. Although the program achieved a notable retention rate of 81.3% at 24 months, the proportion of patients achieving negative urine opioid screens was lower compared to other populations, likely due to concurrent substance use. Our findings underscore the need for MAT programs to adopt a more comprehensive approach that addresses not only opioid use but also the use of other substances at enrolment. Policymakers should consider expanding MAT services and ensuring they are equipped with the necessary resources to manage the full spectrum of substance use disorders, thereby enhancing treatment outcomes.

## Recommendations

To improve the treatment outcomes of Methadone Assisted Treatment (MAT) program, we recommend enhancing resource allocation, implementing integrated treatment strategies for concurrent substance use, optimizing individualized methadone dosing, and developing gender- and age-specific interventions. It is crucial to establish clear guidelines for frequent and consistent drug testing and ensure the availability of testing supplies. Additionally, providing comprehensive care for medical comorbidities, offering socio-economic support, increasing community outreach to reduce stigma, and implementing regular program monitoring and evaluation are critical. Further prospective studies are needed to assess other outcome measures such as relapse rates, mortality, criminality, and general patient quality of life.

## Supporting information

S1 Table Results of bivariate analysis for each concurrent substance used at enrolment, to identify the relationships between concurrent substance use, and socio-demographic and clinical variables. (DOCX)

S1 Data Raw data file. (XLSX)
