# Supplementary material for: Substance use patterns and negative urine opioid screen among patients on methadone treatment at a referral hospital in Nairobi, Kenya
Source: PLOS Ment Health. 2024 Oct 14;1(5):e0000027. doi: 10.1371/journal.pmen.0000027 (PMC12798281; doi:10.1371/journal.pmen.0000027)
Supplement: S1 Table — (DOCX) [file pmen.0000027.s001.docx]

**Factors associated with substance use on Bivariate Analysis.**

| **Variable** | **Category** | **Cannabis** | | | | **Tobacco** | | | | **Alcohol** | | | |
| --- | --- | --- | --- | --- | --- | --- | --- | --- | --- | --- | --- | --- | --- |
|  |  | **No** | **Yes** | **O.R(95% C.I)** | **Sig.** | **No** | **Yes** | **O.R(95% C.I)** | **Sig.** | **No** | **Yes** | **O.R(95% C.I)** | **Sig.** |
| Age | 18-25 | 14(11.5%) | 104(17.6%) | Ref. |  | 13(20.3%) | 105(16.2%) | Ref. |  | 99(17.5%) | 19(12.9%) | Ref. |  |
|  | 26-35 | 50(41.0%) | 262(44.3%) | 0.71(0.37; 1.33) | 0.2810 | 31(48.4%) | 281(43.3%) | 1.12(0.57; 2.23) | 0.7410 | 251(44.3%) | 61(41.5%) | 1.27(0.72; 2.23) | 0.4130 |
|  | 36-50 | 48(39.3%) | 205(34.7%) | 0.58(0.30; 1.09) | 0.0900 | 20(31.3%) | 233(35.9%) | 1.44(0.69; 3.01) | 0.3290 | 191(33.7%) | 62(42.2%) | 1.69(0.96; 2.99) | 0.0700 |
|  | 51 and above | 10(8.2%) | 20(3.4%) | 0.27(0.11; 0.69) | **0.0060** | 0(0.0%) | 30(4.6%) | 1.00(; ) |  | 25(4.4%) | 5(3.4%) | 1.04(0.35; 3.06) | 0.9400 |
| Gender | Female | 19(15.6%) | 83(14.0%) | Ref. |  | 17(26.6%) | 85(13.1%) | Ref. |  | 76(13.4%) | 26(17.7%) | Ref. |  |
|  | Male | 103(84.4%) | 508(86.0%) | 1.13(0.66; 1.94) | 0.6610 | 47(73.4%) | 564(86.9%) | 2.40(1.32; 4.37) | **0.0040** | 490(86.6%) | 121(82.3%) | 0.72(0.44; 1.18) | 0.1900 |
| Education Level | Primary and Below | 57(46.7%) | 291(49.2%) | Ref. |  | 23(35.9%) | 325(50.1%) | Ref. |  | 277(48.9%) | 71(48.3%) | Ref. |  |
|  | Secondary | 54(44.3%) | 236(39.9%) | 0.86(0.57; 1.29) | 0.4570 | 34(53.1%) | 256(39.4%) | 0.53(0.31; 0.93) | **0.0260** | 226(39.9%) | 64(43.5%) | 1.11(0.76; 1.62) | 0.6080 |
|  | Tertiary | 11(9.0%) | 64(10.8%) | 1.14(0.57; 2.30) | 0.7140 | 7(10.9%) | 68(10.5%) | 0.69(0.28; 1.67) | 0.4070 | 63(11.1%) | 12(8.2%) | 0.74(0.38; 1.45) | 0.3850 |
| Marital Status | Married | 24(19.7%) | 130(22.0%) | Ref. |  | 12(18.8%) | 142(21.9%) | Ref. |  | 125(22.1%) | 29(19.7%) | Ref. |  |
|  | Single/Separated/ Widowed | 98(80.3%) | 461(78.0%) | 0.87(0.53; 1.41) | 0.5700 | 52(81.3%) | 507(78.1%) | 0.82(0.43; 1.59) | 0.5620 | 441(77.9%) | 118(80.3%) | 1.15(0.73; 1.81) | 0.5360 |
| Occupation | Employed | 77(63.1%) | 347(58.7%) | Ref. |  | 38(59.4%) | 386(59.5%) | Ref. |  | 337(59.5%) | 87(59.2%) | Ref. |  |
|  | Self-employed | 13(10.7%) | 81(13.7%) | 1.38(0.73; 2.61) | 0.3180 | 4(6.3%) | 90(13.9%) | 2.22(0.77; 6.37) | 0.1400 | 70(12.4%) | 24(16.3%) | 1.32(0.79; 2.23) | 0.2850 |
|  | Un-employed | 32(26.2%) | 163(27.6%) | 1.13(0.72; 1.78) | 0.5960 | 22(34.4%) | 173(26.7%) | 0.77(0.44; 1.35) | 0.3660 | 159(28.1%) | 36(24.5%) | 0.87(0.57; 1.35) | 0.5510 |
| Housing | Homeless | 18(14.8%) | 102(17.3%) | Ref. |  | 6(9.4%) | 114(17.6%) | Ref. |  | 96(17.0%) | 24(16.3%) | Ref. |  |
|  | Friends & Relatives | 30(24.6%) | 129(21.8%) | 0.76(0.40; 1.44) | 0.3980 | 25(39.1%) | 134(20.6%) | 0.28(0.11; 0.71) | **0.0070** | 131(23.1%) | 28(19.0%) | 0.86(0.47; 1.57) | 0.6120 |
|  | Own House/ Rents | 37(30.3%) | 201(34.0%) | 0.96(0.52; 1.77) | 0.8920 | 15(23.4%) | 223(34.4%) | 0.78(0.30; 2.07) | 0.6210 | 189(33.4%) | 49(33.3%) | 1.04(0.60; 1.79) | 0.8960 |
|  | Unstable | 37(30.3%) | 159(26.9%) | 0.76(0.41; 1.40) | 0.3790 | 18(28.1%) | 178(27.4%) | 0.52(0.20; 1.35) | 0.1790 | 150(26.5%) | 46(31.3%) | 1.23(0.70; 2.14) | 0.4710 |
| Legal History | No | 37(30.3%) | 218(36.9%) | Ref. |  | 40(62.5%) | 215(33.1%) | Ref. |  | 199(35.2%) | 56(38.1%) | Ref. |  |
|  | Yes | 85(69.7%) | 373(63.1%) | 0.75(0.49; 1.13) | 0.1700 | 24(37.5%) | 434(66.9%) | 3.36(1.98; 5.73) | **0.0000** | 367(64.8%) | 91(61.9%) | 0.88(0.61; 1.28) | 0.5080 |
| Age at first use | <18 | 23(18.9%) | 125(21.2%) | Ref. |  | 12(18.8%) | 136(21.0%) | Ref. |  | 121(21.4%) | 27(18.4%) | Ref. |  |
|  | 18-25 | 70(57.4%) | 326(55.2%) | 0.86(0.51; 1.43) | 0.5560 | 42(65.6%) | 354(54.5%) | 0.74(0.38; 1.46) | 0.3870 | 314(55.5%) | 82(55.8%) | 1.17(0.72; 1.90) | 0.5230 |
|  | 25+ | 29(23.8%) | 140(23.7%) | 0.89(0.49; 1.62) | 0.6980 | 10(15.6%) | 159(24.5%) | 1.40(0.59; 3.35) | 0.4460 | 131(23.1%) | 38(25.9%) | 1.30(0.75; 2.26) | 0.3510 |
| Duration of use | <8 Years | 34(27.9%) | 224(37.9%) | Ref. |  | 25(39.1%) | 233(35.9%) | Ref. |  | 210(37.1%) | 48(32.7%) | Ref. |  |
|  | 8-15 yrs | 44(36.1%) | 203(34.3%) | 0.70(0.43; 1.14) | 0.1510 | 26(40.6%) | 221(34.1%) | 0.91(0.51; 1.63) | 0.7550 | 194(34.3%) | 53(36.1%) | 1.20(0.77; 1.85) | 0.4230 |
|  | >15 yrs | 44(36.1%) | 164(27.7%) | 0.57(0.35; 0.92) | **0.0230** | 13(20.3%) | 195(30.0%) | 1.61(0.80; 3.23) | 0.1810 | 162(28.6%) | 46(31.3%) | 1.24(0.79; 1.96) | 0.3480 |
| Route of Administration | Intravenous | 86(70.5%) | 312(52.8%) | Ref. |  | 21(32.8%) | 377(58.1%) | Ref. |  | 327(57.8%) | 71(48.3%) | Ref. |  |
|  | Smoking | 18(14.8%) | 140(23.7%) | 2.14(1.24; 3.70) | **0.0060** | 20(31.3%) | 138(21.3%) | 0.38(0.20; 0.73) | **0.0040** | 114(20.1%) | 44(29.9%) | 1.78(1.15; 2.74) | **0.0090** |
|  | Both | 18(14.8%) | 139(23.5%) | 2.13(1.23; 3.67) | **0.0070** | 23(35.9%) | 134(20.6%) | 0.33(0.17; 0.61) | **0.0000** | 125(22.1%) | 32(21.8%) | 1.18(0.74; 1.88) | 0.4880 |
| Maximum Dose | <=50 | 10(8.2%) | 39(6.6%) | Ref. |  | 9(14.1%) | 40(6.2%) | Ref. |  | 44(7.8%) | 5(3.4%) | Ref. |  |
|  | 50-100 | 32(26.2%) | 185(31.3%) | 1.48(0.67; 3.27) | 0.3290 | 21(32.8%) | 196(30.2%) | 2.10(0.90; 4.92) | 0.0880 | 159(28.1%) | 58(39.5%) | 3.21(1.21; 8.49) | **0.0190** |
|  | 100-150 | 58(47.5%) | 250(42.3%) | 1.11(0.52; 2.34) | 0.7940 | 23(35.9%) | 285(43.9%) | 2.79(1.21; 6.45) | **0.0170** | 242(42.8%) | 66(44.9%) | 2.40(0.92; 6.29) | 0.0750 |
|  | 151- 200 | 16(13.1%) | 100(16.9%) | 1.60(0.67; 3.84) | 0.2890 | 10(15.6%) | 106(16.3%) | 2.39(0.90; 6.30) | 0.0790 | 100(17.7%) | 16(10.9%) | 1.41(0.49; 4.08) | 0.5290 |
|  | >200 | 6(4.9%) | 17(2.9%) | 0.73(0.23; 2.32) | 0.5900 | 1(1.6%) | 22(3.4%) | 4.95(0.59; 41.67) | 0.1410 | 21(3.7%) | 2(1.4%) | 0.84(0.15; 4.68) | 0.8410 |

| **Variable** | **Category** | **Cocaine** | | | | **Benzodiazepines** | | | | **Khat** | | | | **Benzhexol** | | | |
| --- | --- | --- | --- | --- | --- | --- | --- | --- | --- | --- | --- | --- | --- | --- | --- | --- | --- |
|  |  | **No** | **Yes** | **O.R(95% C.I)** | **Sig.** | **No** | **Yes** | **O.R(95% C.I)** | **Sig.** | **No** | **Yes** | **O.R(95% C.I)** | **Sig.** | **No** | **Yes** | **O.R(95% C.I)** | **Sig.** |
| Age | 18-25 | 117(16.9%) | 1(5.3%) | Ref. |  | 51(14.7%) | 67(18.3%) | Ref. |  | 106(16.6%) | 12(15.8%) | Ref. |  | 111(16.4%) | 7(20.0%) | Ref. |  |
|  | 26-35 | 303(43.7%) | 9(47.4%) | 3.48(0.44; 27.73) | 0.2400 | 149(43.1%) | 163(44.4%) | 0.83(0.54; 1.28) | 0.4000 | 276(43.3%) | 36(47.4%) | 1.15(0.58; 2.30) | 0.6880 | 299(44.1%) | 13(37.1%) | 0.69(0.27; 1.77) | 0.4400 |
|  | 36-50 | 244(35.2%) | 9(47.4%) | 4.32(0.54; 34.47) | 0.1680 | 124(35.8%) | 129(35.1%) | 0.79(0.51; 1.23) | 0.2980 | 227(35.6%) | 26(34.2%) | 1.01(0.49; 2.08) | 0.9750 | 240(35.4%) | 13(37.1%) | 0.86(0.33; 2.21) | 0.7530 |
|  | 51 and above | 30(4.3%) | 0(0.0%) | 1.00(; ) |  | 22(6.4%) | 8(2.2%) | 0.28(0.11; 0.67) | **0.0050** | 28(4.4%) | 2(2.6%) | 0.63(0.13; 2.98) | 0.5610 | 28(4.1%) | 2(5.7%) | 1.13(0.22; 5.75) | 0.8810 |
| Gender | Female | 98(14.1%) | 4(21.1%) | Ref. |  | 52(15.0%) | 50(13.6%) | Ref. |  | 83(13.0%) | 19(25.0%) | Ref. |  | 100(14.7%) | 2(5.7%) | Ref. |  |
|  | Male | 596(85.9%) | 15(78.9%) | 0.62(0.20; 1.90) | 0.3990 | 294(85.0%) | 317(86.4%) | 1.12(0.74; 1.71) | 0.5920 | 554(87.0%) | 57(75.0%) | 0.45(0.26; 0.79) | **0.0060** | 578(85.3%) | 33(94.3%) | 2.86(0.67; 12.09) | 0.1540 |
| Education Level | Primary and Below | 342(49.3%) | 6(31.6%) | Ref. |  | 172(49.7%) | 176(48.0%) | Ref. |  | 310(48.7%) | 38(50.0%) | Ref. |  | 329(48.5%) | 19(54.3%) | Ref. |  |
|  | Secondary | 281(40.5%) | 9(47.4%) | 1.83(0.64; 5.20) | 0.2590 | 143(41.3%) | 147(40.1%) | 1.01(0.746 1.37) | 0.9770 | 264(41.4%) | 26(34.2%) | 0.80(0.48; 1.36) | 0.4140 | 275(40.6%) | 15(42.9%) | 0.94(0.47; 1.89) | 0.8720 |
|  | Tertiary | 71(10.2%) | 4(21.1%) | 3.21(0.88; 11.67) | 0.0760 | 31(9.0%) | 44(12.0%) | 1.39(0.84; 2.30) | 0.2040 | 63(9.9%) | 12(15.8%) | 1.55(0.77; 3.14) | 0.2190 | 74(10.9%) | 1(2.9%) | 0.23(0.03; 1.78) | 0.1600 |
| Marital Status | Married | 150(21.6%) | 4(21.1%) | Ref. |  | 88(25.4%) | 66(18.0%) | Ref. |  | 140(22.0%) | 14(18.4%) | Ref. |  | 149(22.0%) | 5(14.3%) | Ref. |  |
|  | Single/Separated/ Widowed | 544(78.4%) | 15(78.9%) | 1.03(0.34; 3.16) | 0.9530 | 258(74.6%) | 301(82.0%) | 1.56(1.09; 2.23) | **0.0160** | 497(78.0%) | 62(81.6%) | 1.25(0.68; 2.30) | 0.4770 | 529(78.0%) | 30(85.7%) | 1.69(0.64; 4.43) | 0.2860 |
| Occupation | Employed | 412(59.4%) | 12(63.2%) | Ref. |  | 212(61.3%) | 212(57.8%) | Ref. |  | 383(60.1%) | 41(53.9%) | Ref. |  | 407(60.0%) | 17(48.6%) | Ref. |  |
|  | Self-employed | 90(13.0%) | 4(21.1%) | 1.53(0.48; 4.84) | 0.4730 | 46(13.3%) | 48(13.1%) | 1.04(0.67; 1.63) | 0.8520 | 81(12.7%) | 13(17.1%) | 1.50(0.77; 2.93) | 0.2350 | 92(13.6%) | 2(5.7%) | 0.52(0.12; 2.29) | 0.3880 |
|  | Un-employed | 192(27.7%) | 3(15.8%) | 0.54(0.15; 1.92) | 0.3390 | 88(25.4%) | 107(29.2%) | 1.22(0.87; 1.71) | 0.2600 | 173(27.2%) | 22(28.9%) | 1.19(0.69; 2.06) | 0.5380 | 179(26.4%) | 16(45.7%) | 2.14(1.06; 4.33) | **0.0340** |
| Housing | Homeless | 118(17.0%) | 2(10.5%) | Ref. |  | 62(17.9%) | 58(15.8%) | Ref. |  | 108(17.0%) | 12(15.8%) | Ref. |  | 113(16.7%) | 7(20.0%) | Ref. |  |
|  | Friends & Relatives | 154(22.2%) | 5(26.3%) | 1.92(0.37; 10.05) | 0.4420 | 68(19.7%) | 91(24.8%) | 1.43(0.89; 2.30) | 0.1410 | 140(22.0%) | 19(25.0%) | 1.22(0.57; 2.63) | 0.6080 | 149(22.0%) | 10(28.6%) | 1.08(0.40; 2.93) | 0.8750 |
|  | Own House/ Rents | 232(33.4%) | 6(31.6%) | 1.53(0.30; 7.68) | 0.6080 | 129(37.3%) | 109(29.7%) | 0.90(0.58; 1.40) | 0.6500 | 210(33.0%) | 28(36.8%) | 1.20(0.59; 2.45) | 0.6170 | 233(34.4%) | 5(14.3%) | 0.35(0.11; 1.12) | 0.0760 |
|  | Unstable | 190(27.4%) | 6(31.6%) | 1.86(0.37; 9.38) | 0.4510 | 87(25.1%) | 109(29.7%) | 1.34(0.85; 2.11) | 0.2090 | 179(28.1%) | 17(22.4%) | 0.86(0.39; 1.86) | 0.6920 | 183(27.0%) | 13(37.1%) | 1.15(0.44; 2.96) | 0.7770 |
| Legal History | No | 249(35.9%) | 6(31.6%) | Ref. |  | 122(35.3%) | 133(36.2%) | Ref. |  | 216(33.9%) | 39(51.3%) | Ref. |  | 244(36.0%) | 11(31.4%) | Ref. |  |
|  | Yes | 445(64.1%) | 13(68.4%) | 1.21(0.46; 3.23) | 0.7000 | 224(64.7%) | 234(63.8%) | 0.96(0.71; 1.30) | 0.7850 | 421(66.1%) | 37(48.7%) | 0.49(0.302; 0.79) | **0.0030** | 434(64.0%) | 24(68.6%) | 1.23(0.59; 2.55) | 0.5840 |
| Age at first use | <18 | 140(20.2%) | 8(42.1%) | Ref. |  | 58(16.8%) | 90(24.5%) | Ref. |  | 135(21.2%) | 13(17.1%) | Ref. |  | 139(20.5%) | 9(25.7%) | Ref. |  |
|  | 18-25 | 387(55.8%) | 9(47.4%) | 0.41(0.15; 1.08) | 0.0700 | 195(56.4%) | 201(54.8%) | 0.66(0.45; 0.98) | **0.0370** | 348(54.6%) | 48(63.2%) | 1.43(0.75; 2.73) | 0.2740 | 379(55.9%) | 17(48.6%) | 0.69(0.30; 1.59) | 0.3870 |
|  | 25+ | 167(24.1%) | 2(10.5%) | 0.21(0.04; 1.00) | **0.0500** | 93(26.9%) | 76(20.7%) | 0.53(0.34; 0.82) | **0.0050** | 154(24.2%) | 15(19.7%) | 1.01(0.47; 2.20) | 0.9770 | 160(23.6%) | 9(25.7%) | 0.87(0.34; 2.25) | 0.7720 |
| Duration of use | <8 Years | 255(36.7%) | 3(15.8%) | Ref. |  | 127(36.7%) | 131(35.7%) | Ref. |  | 227(35.6%) | 31(40.8%) | Ref. |  | 246(36.3%) | 12(34.3%) | Ref. |  |
|  | 8-15 yrs | 239(34.4%) | 8(42.1%) | 2.85(0.75; 10.85) | 0.1260 | 121(35.0%) | 126(34.3%) | 1.01(0.71; 1.43) | 0.9580 | 219(34.4%) | 28(36.8%) | 0.94(0.54; 1.61) | 0.8120 | 237(35.0%) | 10(28.6%) | 0.87(0.37; 2.04) | 0.7400 |
|  | >15 yrs | 200(28.8%) | 8(42.1%) | 3.40(0.89; 12.98) | 0.0730 | 98(28.3%) | 110(30.0%) | 1.09(0.76; 1.57) | 0.6510 | 191(30.0%) | 17(22.4%) | 0.65(0.35; 1.21) | 0.1770 | 195(28.8%) | 13(37.1%) | 1.37(0.61; 3.06) | 0.4480 |
| Route of opioid Administration | Intravenous | 384(55.3%) | 14(73.7%) | Ref. |  | 186(53.8%) | 212(57.8%) | Ref. |  | 364(57.1%) | 34(44.7%) | Ref. |  | 371(54.7%) | 27(77.1%) | Ref. |  |
|  | Smoking | 155(22.3%) | 3(15.8%) | 0.53(0.15; 1.87) | 0.3250 | 83(24.0%) | 75(20.4%) | 0.79(0.55; 1.15) | 0.2180 | 135(21.2%) | 23(30.3%) | 1.82(1.04; 3.21) | **0.0370** | 154(22.7%) | 4(11.4%) | 0.36(0.12; 1.04) | 0.0580 |
|  | Both | 155(22.3%) | 2(10.5%) | 0.35(0.08; 1.58) | 0.1730 | 77(22.3%) | 80(21.8%) | 0.91(0.63; 1.32) | 0.6230 | 138(21.7%) | 19(25.0%) | 1.47(0.81; 2.67) | 0.2010 | 153(22.6%) | 4(11.4%) | 0.36(0.12; 1.04) | 0.0600 |
| Maximum Dose | <=50 | 46(6.6%) | 3(15.8%) | Ref. |  | 20(5.8%) | 29(7.9%) | Ref. |  | 44(6.9%) | 5(6.6%) | Ref. |  | 48(7.1%) | 1(2.9%) | Ref. |  |
|  | 50-100 | 210(30.3%) | 7(36.8%) | 0.51(0.13; 2.05) | 0.3440 | 103(29.8%) | 114(31.1%) | 0.76(0.41; 1.43) | 0.4000 | 197(30.9%) | 20(26.3%) | 0.89(0.32; 2.51) | 0.8310 | 207(30.5%) | 10(28.6%) | 2.32(0.290; 18.55) | 0.4280 |
|  | 100-150 | 303(43.7%) | 5(26.3%) | 0.25(0.06; 1.10) | 0.0660 | 152(43.9%) | 156(42.5%) | 0.71(0.38; 1.31) | 0.2680 | 271(42.5%) | 37(48.7%) | 1.20(0.45; 3.22) | 0.7150 | 289(42.6%) | 19(54.3%) | 3.16(0.41; 24.12) | 0.2680 |
|  | 151- 200 | 113(16.3%) | 3(15.8%) | 0.41(0.08; 2.09) | 0.2820 | 62(17.9%) | 54(14.7%) | 0.60(0.31; 1.18) | 0.1400 | 105(16.5%) | 11(14.5%) | 0.92(0.30; 2.81) | 0.8860 | 112(16.5%) | 4(11.4%) | 1.71(0.19; 15.74) | 0.6340 |
|  | >200 | 22(3.2%) | 1(5.3%) | 0.70(0.07; 7.09) | 0.7600 | 9(2.6%) | 14(3.8%) | 1.07(0.39; 2.95) | 0.8920 | 20(3.1%) | 3(3.9%) | 1.32(0.29; 6.07) | 0.7210 | 22(3.2%) | 1(2.9%) | 2.18(0.13; 36.51) | 0.5870 |
